# Supplementary material for: Modelling the Distribution of Cognitive Outcomes for Early-Stage Neurocognitive Disorders: A Model Comparison Approach
Source: Biomedicines. 2024 Feb 8;12(2):393. doi: 10.3390/biomedicines12020393 (PMC10886528; doi:10.3390/biomedicines12020393)
Supplement: Supplementary file 1 [file biomedicines-12-00393-s001.zip › biomedicines-2797210-supplementary.pdf]

Supplement Table S1. Model's performance, MoCA and inverse MoCA outcome scores

Outcome variable	Distribution	Intercept-only Model			Model with covariates		
		-2LL	AIC	BIC	-2LL	AIC	BIC
MoCA	Normal	4977.2	4981.2	4990.7	4849.8	4859.8	4883.8
	Log-Normal	5431.0	5435.0	5444.6	5319.2	5329.2	5353.1
	Gamma	5258.3	5262.3	5271.8	5129.8	5139.8	5163.7
	Poisson	5133.5	5135.5	5140.2	5053.7	5061.7	5060.8
	NB	5134.6	5138.6	5148.2	5062.0	5072.0	5095.9
	CMP	5094.1	5098.1	5107.7	4978.0	4988.0	5011.9
	GP	5134.6	5138.6	5148.2	5080.8	5090.8	5114.7
	Weibull	4705.0	4709.0	4718.6	4603.8	4613.8	4637.7
Inverse MoCA	Normal	4977.2	4981.2	4990.7	4849.8	4859.8	4883.8
	Log-Normal	4418.7	4422.7	4432.3	4306.5	4316.5	4340.4
	Gamma	4470.0	4474.0	4483.6	4356.5	4366.5	4390.4
	Poisson	5395.5	5397.5	5402.3	5039.9	5047.9	5067.0
	NB	4457.1	4461.1	4470.7	4348.2	4358.2	4382.1
	CMP	4494.1	4498.1	4507.6	4386.9	4396.9	4420.8
	GP	4440.1	4444.1	4453.6	4333.9	4343.9	4367.9
	Weibull	4537.0	4541.0	4550.5	4501.8	4511.8	4535.7

Abbreviations: MoCA = Montreal Cognitive Assessment; LL = log-likelihood; AIC = Akaike's Information Criterion; BIC = Bayesian Information Criterion; NB = Negative Binomial; CMP = Conway-Maxwell-Poisson; GP = Generalized Poisson

Supplement Table S2: Association analysis of the baseline characteristics with MoCA and inverse (MoCA) outcomes

Model	Variable	MoCA Score		Inverse MoCA Score	
		Beta (95% CI)	P value	Beta (95% CI)	P value
CMP	Age (year)	0.004 (-0.015, 0.023)	NS	0.003 (-0.017, 0.024)	NS
	Female Gender	0.088 (0.051, 0.124)	<.0001	-0.103 (-0.147, -0.059)	<.0001
	Education (year)	0.095 (0.077, 0.113)	<.0001	-0.07 (-0.072, -0.068)	<.0001
Gamma	Age (year)	0.239 (-0.151, 0.628)	NS	0.024 (-0.073, 0.121)	NS
	Female Gender	2.242 (1.42, 3.063)	<.0001	-0.544 (-0.757, -0.33)	<.0001
	Education (year)	2.059 (1.657, 2.461)	<.0001	-0.589 (-0.723, -0.454)	<.0001
GP	Age (year)	-0.008 (-0.022, 0.005)	NS	0.032 (-0.022, 0.086)	NS
	Female Gender	0.023 (-0.004, 0.051)	NS	-0.257 (-0.361, -0.154)	<.0001
	Education (year)	0.06 (0.045, 0.075)	<.0001	-0.212 (-0.25, -0.174)	<.0001
LogNormal	Age (year)	0.008 (-0.005, 0.022)	NS	0.021 (-0.024, 0.065)	NS
	Female Gender	0.071 (0.044, 0.099)	<.0001	-0.245 (-0.337, -0.153)	<.0001
	Education (year)	0.071 (0.057, 0.084)	<.0001	-0.222 (-0.267, -0.177)	<.0001
Poisson	Age (year)	0.002 (-0.011, 0.016)	NS	0.004 (-0.029, 0.036)	NS
	Female Gender	0.057 (0.029, 0.085)	<.0001	-0.298 (-0.363, -0.232)	<.0001
	Education (year)	0.061 (0.047, 0.076)	<.0001	-0.228 (-0.252, -0.205)	<.0001
NB	Age (year)	0.003 (NE)	-	0.026 (-0.027, 0.08)	NS
	Female Gender	0.057 (NE)	-	-0.263 (-0.367, -0.16)	<.0001
	Education (year)	0.062 (NE)	-	-0.214 (-0.251, -0.176)	<.0001
Normal	Age (year)	0.047 (-0.212, 0.306)	NS	-0.047 (-0.306, 0.212)	NS
	Female Gender	1.42 (0.888, 1.952)	<.0001	-1.42 (-1.95, -0.888)	<.0001
	Education (year)	1.448 (1.186, 1.71)	<.0001	-1.45 (-1.71, -1.19)	<.0001

Weibull	Age (year)	0.385 (-0.138, 0.909)	NS	0.104 (0.041, 0.167)	0.0013
	Female Gender	2.254 (1.314, 3.195)	<.0001	0.179 (0.041, 0.317)	0.0109
	Education (year)	1.463 (1.284, 1.643)	<.0001	0.139 (0.09, 0.189)	<.0001

Abbreviations: MoCA = Montreal Cognitive Assessment; NB = Negative Binomial; CMP = Conway-Maxwell-Poisson; GP = Generalized Poisson; NS = Not Significant; NE = Not Estimable

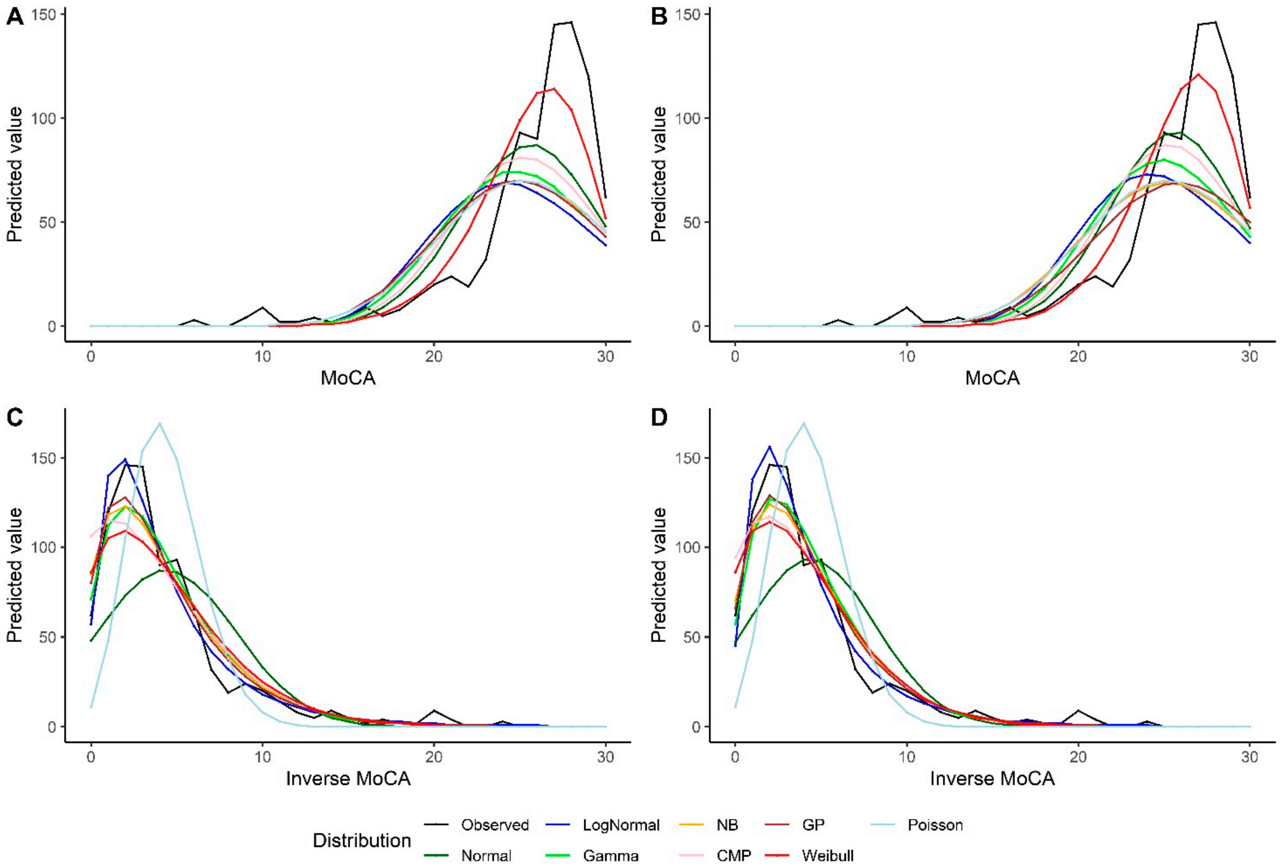

Fitted values by various distributions under different scenarios: A) without covariates, original score; B) with covariates (with age, gender and education variables as predictors), original score C) without covariates, inverse score; D) with covariates (with age, gender and education variables as predictors), inverse score.

Supplement Figure S1: Predicted values among the fitted models, MoCA and Inverse MoCA outcomes

Supplement Table S3: Probability mass functions of the distributions

A concise review of the relevant probability distributions used in this paper:

Normal: The random variable Y follows the normal distribution with mean μ and variance σ^2 if its probability density function is given by

$$f_Y(y;\mu,\sigma^2) = \frac{1}{\sqrt{2\pi\sigma^2}} e^{-\frac{1}{2\sigma^2}(y-\mu)^2}, y \in \mathbb{R}, \mu \in \mathbb{R}, \sigma > 0.$$

Log-Normal: The random variable Y has the log-normal distribution if its probability density function can be written as

$$f_Y(y;\mu,\sigma^2) = \frac{1}{y\sqrt{2\pi\sigma^2}} e^{\frac{-1}{2\sigma^2}(ln(y)-\mu)^2}, y > 0, \mu \in \mathbb{R}, \sigma > 0.$$

The mean and variance of Y are $E(Y) = e^{\left(\mu+\frac{\sigma^2}{2}\right)}$, and $Var(Y) = \left(e^{\sigma^2} - 1\right)e^{(2\mu+\sigma^2)}$, respectively.

Gamma: The random variable Y follows the gamma distribution with shape parameter $k > 0$ and scale parameter $\theta > 0$, if its probability density function is equal to

$$f_Y(y;k,\theta)=\frac{1}{\Gamma(k)\theta^k}y^{k-1}e^{-\frac{y}{\theta}}, y>0.$$

The mean and variance of Y are $E(Y)=k\theta$, $Var(Y)=k\theta^2$.

Weibull: The random variable Y follows the Weibull distribution with the scale parameter λ and the shape parameter k, when its probability density function is given by

$$f_Y(y;k,\lambda)=\frac{k}{\lambda}\left(\frac{y}{\lambda}\right)^{k-1}e^{-\left(\frac{y}{\lambda}\right)^k}, y>0, \lambda>0, k>0.$$

Mean and variance of Y are $E(Y)=\lambda\Gamma\left(1+\frac{1}{k}\right)$, and $Var(Y)=\lambda^2\left[\Gamma\left(1+\frac{2}{k}\right)-\left(\Gamma\left(1+\frac{1}{k}\right)\right)^2\right]$.

Poisson: The random variable Y follows the Poisson distribution if its probability mass function is given by

$$P(Y=y)=\frac{e^{-\lambda}\lambda^y}{y!}, y\in\{0,1,2,\dots\}, \lambda>0.$$

Both mean and variance of Poisson distribution are equal to λ.

Negative Binomial: The random variable Y as the number of failures until a predefined number of successes, indicating by r, occurs follows the negative binomial distribution when its probability mass function is given by

$$P(Y=y)=\binom{r+y-1}{y}p^r(1-p)^y, y\in\{0,1,2,\dots\},$$

where $0<p<1$ is the probability of success. The mean and variance of Y are $E(Y)=\frac{r(1-p)}{p}$, and $Var(Y)=\frac{r(1-p)}{p^2}$, respectively.

Conway-Maxwell-Poisson: The random variable Y follows the Conway-Maxwell-Poisson if its probability mass function can be written by

$$P(Y=y)=\frac{\lambda^y}{(y!)^v}\frac{1}{Z(\lambda,v)}, y\in\{0,1,2,\dots\}, \lambda>0, v\geq 0,$$

where $Z(\lambda,v)=\sum_{j=0}^{\infty}\frac{\lambda^j}{(j!)^v}$.

Mean and variance of Y are $E(Y)=\sum_{j=0}^{\infty}\frac{j\lambda^j}{(j!)^v Z(\lambda,v)}$, and $Var(Y)=\sum_{j=0}^{\infty}\frac{j^2\lambda^j}{(j!)^v Z(\lambda,v)}-\left(\sum_{j=0}^{\infty}\frac{j\lambda^j}{(j!)^v Z(\lambda,v)}\right)^2$.

Generalized Poisson: The random variable Y follows the generalized Poisson distribution when its probability mass function is equal to

$$P(Y=y)=\frac{\lambda}{y!}(\lambda+\alpha y)^{y-1}e^{-(\lambda+\alpha y)}, y\in\{0,1,2,\dots\},$$

where $\lambda>0$ and $0\leq\alpha<1$ (Joe and Zhu, 2005). Mean and variance of Y are $E(Y)=\frac{\lambda}{(1-\alpha)}$, and $Var(Y)=\frac{\lambda}{(1-\alpha)^3}$, respectively.
